# New method of producing a more efficient coagulant for the treatment of water from seeds of moringa oleifera

**DOI:** 10.1016/j.mex.2023.102485

**Published:** 2023-11-16

**Authors:** Parfait Sagnon Hounsinou, Fidèle Mahoudo Assogba, Miriam Hounsinou, Julien Adounkpè, Lyde Sèwèdo Arsène Tomètin, Achille Comlan Dedjiho, Waris Kéwouyèmi Chouti, Daouda Mama, Joachim Djimon Gbénou, Eléonore Yayi Ladekan

**Affiliations:** a Faculté des Sciences et Technique (FAST), Université d'Abomey – Calavi (UAC), Bénin; b Laboratoire de Pharmacognosie et des Huiles Essentielles, 01 BP: 918, ISBA, Cotonou, Bénin; c Institut de Mathématiques et de Sciences Physiques, Université d'Abomey-Calavi (UAC), Bénin; d Laboratoire de Physique du Rayonnement, Faculté des Sciences et Techniques (FAST), Université d'Abomey-Calavi (UAC), Bénin; e Département de l'Aménagement et de la Gestion de l'Environnement, Faculté des sciences agronomiques (FSA), Université d'Abomey-Calavi (UAC), Bénin; f Laboratoire KABA de Chimie et Applications, Université Nationale des Sciences, Technologies, Ingénierie et Mathématiques (UNSTIM), Abomey BP 486, Bénin; g Laboratoire de chimie inorganique et de l'environnement, Bénin; h Laboratoire de Chimie Physique, Matériaux et Modélisation Moléculaire, Bénin; i Laboratoire d'Hydrologie Appliquée (LHA), Institut Nationale de l'Eau (INE), Université d'Abomey-Calavi (UAC), Cotonou 05 BP 9148, Bénin

**Keywords:** Organic chemistry, Extraction, Coagulant, Water treatment, None

## Abstract

The coagulation is essential in raw water treatment. The most used coagulants are often of chemical origin and expensive and their use generates non-biodegradable residues. This is why innovative studies on the synthesis of natural coagulants based on plant extracts are very important. This study presents a new method of producing a coagulant for the treatment of water from seeds of *moringa oleifera*. The application of the new method is done in several steps: Harvesting moringa oleifera seeds, shelling, crushing the seeds, extracting oil from moringa oleifera powder, extracting salts from powder from moringa seeds oleifera, microfiltration with a 0.2 µm filter and lyophilization which gives the final product in dry form. We used the resulting product for coagulation of lake water with an initial turbidity of 40 NTU. This treatment reduced the turbidity of the water by almost 95 % when we used 0.7 mg of this coagulant per liter of water. Moringa has previously been used to produce coagulant but the peculiarity of this study is that it takes a very small amount of the synthesized product to effectively treat water.

­ The results of this research work have shown that the coagulant produced rom the seeds of Moringa oleifera can be used effectively for the treatment of surface water.
­ The residual turbidity obtained after the treatment of lake water with the coagulant produced was in accordance with the drinking water standard according to the World Health Organization (WHO) (less than 5 NTU).
­ The coagulant produced by this new method from moringa oleifera seed is an alternative to aluminum sulfate coagulant for water treatment.

## Graphical abstract

The steps of the new method of producing of coagulant for the treatment of water from seeds of moringa oleifera:

**Figure:**
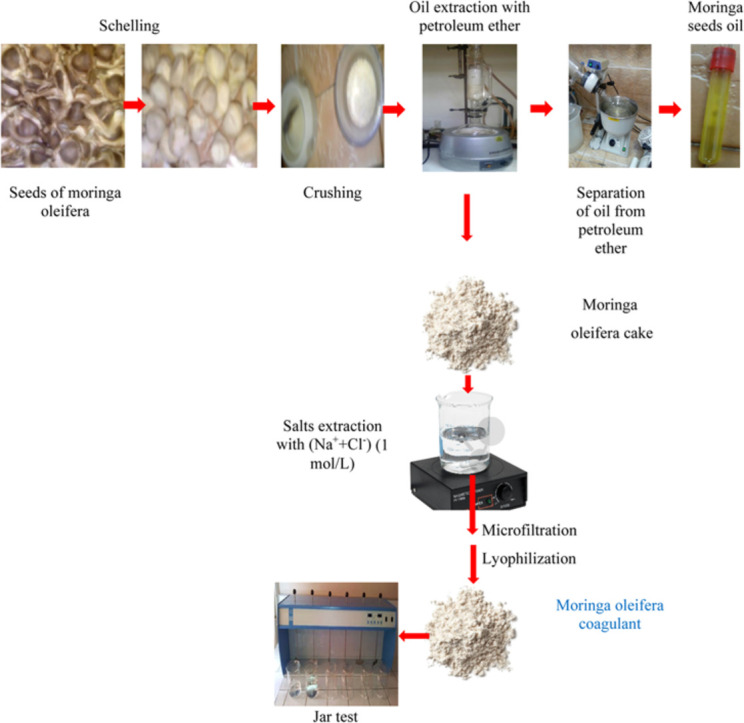

Specifications table

**Table:** 

Subject area:	Environmental Science
More specific subject area:	*Water treatment*
Name of your method:	None
Name and reference of original method:	None
Resource availability:	Soxhlet electro thermal, shredder, petroleum ether, molar aqueous solution of sodium chloride, magnetic stirrer, 0.2 µm filter, jar test device.

## Introduction

Groundwater is abundant and minimally polluted and is often used to produce drinking water [[1], [2], [3]]. The coagulation is the first treatment for water. It brings together the elements in suspension in the water which are then removed by decantation or filtration. The product most often used for coagulation in water treatment is alum or aluminum sulfate (Al_4_SO_3_). But the use of aluminum sulfate in water treatment makes the treated water acidic and overloaded with aluminum ions. To return the pH of the treated water to normal, lime is added to it, which increases the overall treatment cost of the water [4]. In particular, during wastewater treatment, chemical coagulants generate non-biodegradable sludge residues [5] which present health problems and environmental impact [6,7].

Previous studies have shown that from moringa and in particular from moringa oleifera can produce a good coagulant for water treatment.

Some researchers extracted only the oil from the seeds of moringa to improve its coagulation power and the cakes of the seeds of moringa are used to carry out the coagulation of the water [8], [9], [10].

Other research has made it possible to extract the coagulant from moringa seeds using only saline solutions. Aqueous solutions of NaCl, HCl, KCl, NaNO_3_, NaOH, NH_4_Cl and KNO_3_ are saline solutions and are most used to extract natural coagulants [11,12].

These studies have shown that the doses of coagulant used to clarify water with initial turbidity greater than or equal to around forty NTU based on coagulant from moringa seeds extracted only with a saline solution or extracted only with soxhlet are high (greater than 35 mg/L). For example, this year, a study improved, the removal of maximum turbidity from wastewater from a dairy industry with high initial turbidity (614 NTU) to 96.8 % when this wastewater is treated with 300 mg L^−1^ of Moringa seed coagulant extracted with a 0.9 mol L^−1^ CaCl_2_ solution [13]. Although overall, previous studies have found that moringa seed coagulant doses are lower compared to alum doses used to achieve the same water clarification results, alum is still the coagulant the most used. It is necessary to produce a best moringa seeds coagulant for clarifying water at very low doses in order to considerably reduce the cost of water clarification and thus make more economically attractive the use of moringa seeds coagulant which is very ecological.

This study aims to produce a more effective coagulant based on moringa seeds, for example, for treating water with turbidity equal to around forty NTU, with a very low dose of coagulant (less than 1 mg/L).

Indeed, saline solutions are used to extract salts from moringa seeds but they do not allow the oil to be extracted from moringa seeds. However, the oil reduces the coagulating power of moringa seeds. This is the major shortcoming of coagulants extracted from moringa seeds using only saline solutions. Thus, before treating the moringa seeds with a saline solution to rid the moringa seeds of salts, it is important and advantageous to treat the moringa seeds with a soxhlet. The coagulant obtained is therefore more effective.

The objective of the present study is to produce a very effective, ecological and economical coagulant by combining the treatments of moringa seeds by soxhlet and with saline solutions and then application of the coagulant obtained on lake water.

## Method details

### The steps of the production of the coagulant of moringa oleifera seeds

The steps of production used are: harvesting moringa oleifera seeds, shelling, crushing the seeds, extracting oil from moringa oleifera powder, extracting salts from powder from moringa seeds oleifera, microfiltration with a 0.2 µm filter and lyophilization which gives the final product in dry form. This product is used to treat water.

#### Harvesting moringa oleifera seeds

Green colored moringa oleifera seeds are unripe. Green seeds have no coagulation activity [14]. The moringa oleifera seeds used for this study are ripe, dried seeds on trees.

#### Shelling of moringa oleifera seeds

The shelling of the seeds of moringa oleifera was carried out manually.

#### Crushing the seeds

Moringa oleifera seeds are crushed by a grinder.

#### Extracting the oil from the powder of moringa oleifera seeds

The oil extraction was carried out in several phases:

- Weighed 10 g of Moringa oleifera seed powder.
- Introduced this sample into the cartridge of the extraction chamber of the soxhlet electrothermal device.
- 200 ml of petroleum ether were added to the extraction chamber of the soxhlet electrothermal device.
- The petroleum ether was evaporated for a period of 6 h.
- At the end of the oil extraction, the oil was separated from the petroleum ether using a rotary steamer.
- The Moringa oleifera cake was dried.

#### Extraction of salts from the powder of moringa oleifera seeds

0.5 liter of an aqueous solution of sodium chloride of concentration 1 mol/L was introduced into a beaker. 2.5 g of the Moringa oleifera cake was dissolved in the sodium chloride solution and the mixture was stirred for 30 min using a magnetic stirrer. The solution obtained is filtered with filter paper and the solution which has passed through the filter paper is used for the rest of the research.

#### Microfiltration

The aqueous solution of sodium chloride and moringa oleifera cake was filtered through a microfiltration cartridge with pores of 0.20 µm size. The solution which passed through the pores of the microfiltration cartridge was used for the remainder of the study.

#### Lyophilization

Lyophilization of the solution obtained after microfiltration gave a white powder, completely soluble in water. This powder has a very high coagulation activity. The protein concentration of this white powder is measured by the protein assay method [15]. This powder consists almost entirely of protein (99 % protein).

The natural coagulant produced during this study is evaluated by the jar test method. The powder obtained by this study is a high-yield natural coagulant. This coagulant is used to treat lake water with an initial turbidity of 40 NTU. At the same time, other tests were carried out to compare the use of aluminum sulfate (the most common coagulant) and the coagulant produced during this study.

### The steps of the treatment of water with coagulant from moringa oleifera seeds

The equipment consists of a precision balance, saucer, spatula, volumetric flask with capacity *V* = 100 mL, distilled water, water wash bottle, graduated pipette, lake water to clarify, graduated cylinder and test jar device with 6 beakers and a paddle for stirring the water contained in each beaker.

The masses of the moringa oleifera seed coagulant to be used to clarify the water contained in each beaker of the jar test device are very low. The protocol adopted during this study is as follows:

­ Introduce a volume of 0.5 L of lake water of known turbidity into each of the 6 beakers of the test jar device.
­ Prepare a stock coagulant solution by dissolving a mass *m* = 100 mg of the coagulant from moringa oleifera seeds in a volume *V* = 100 mL of distilled water.
­ Take a volume V_0_ = 10 mL of the stock coagulant solution and add a volume equal to 9V_0_ of distilled water to obtain a 1/10^th^ dilution of the stock coagulant solution.
­ Take a volume V_0_ of the solution diluted to 1/10^th^ of the coagulant stock solution and add a volume equal to 9V_0_ of distilled water to obtain a 1/100^th^ dilution of the coagulant stock solution.
­ Using a graduated pipette, introduce into each of the 2^nd^ to 6^th^ beakers of the test jar device a volume (V_1_) of stock coagulant solution and/or a volume (V_2_) of its 1/10^th^ dilution and /or a volume (V_3_) of its dilution at 1/100^th^ in order to have the dose (D) of desired coagulant in the water to clarify. The lake water contained in the first beaker of the jar test device plays the role of control.

*D* = (m ÷ V) (2V_1_ + V_2_ ÷ 5 + V_3_ ÷ 50) where V, V_1_, V_2_ and V_3_ are in mL, m is in mg and D is in mg/L.

- Stir the mixture of water to be clarified and the coagulant contained in each beaker of the test jar device for 30 min then leave the mixtures to rest for 1 hour.

**Fig. 1:**
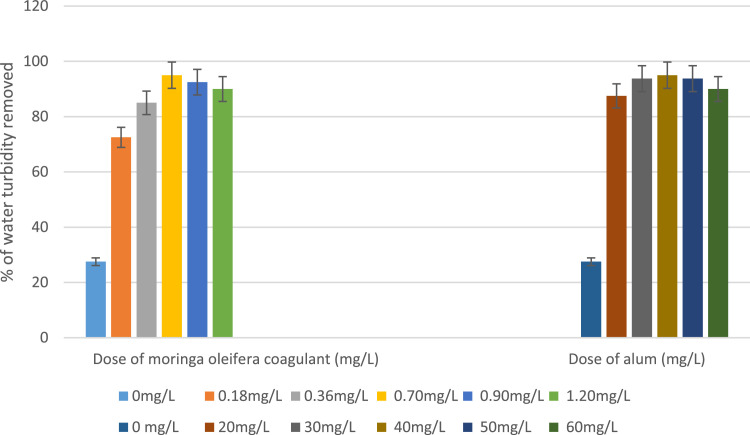
% of turbidity removed of lake water treated with the coagulant of *moringa oleifera* seeds and with alum.

The average doses of coagulant introduced into the 6 beakers of the jar test device are equal to 0, 0.18 mg/L, 0.36 mg/L, 0.70 mg/L, 0.90 mg/L, 1.20 mg/L and the percentages of reduction of turbidity of clarified water were respectively on average equal to 27.5 %, 72.5 %, 85, 95 %, 92.5 %, 90 % (Fig. 1).

The dose that allowed the greatest reduction in turbidity is on average 0.7 mg/L.

## Comparison of the coagulant of moringa oleifera seeds to aluminum sulfate

The equipment is the same as that used to clarify lake water using the coagulant from moringa oleifera seeds. The steps of lake water clarification are as follows:

- Introduce a volume of 0.5 L of lake water of known turbidity into each of the 6 beakers of the test jar device.
- Prepare a coagulant solution by dissolving a mass *m* = 200 mg of aluminum sulfate in a volume *V* = 100 mL of distilled water. It is not necessary to dilute this coagulant solution.
- Using a graduated pipette, introduce into each of the 2^nd^ to 6^th^ beakers of the test jar device a volume (V_1_) of coagulant solution in order to have the dose (D) of coagulant in the water to be clarified wish. The lake water contained in the first beaker of the jar test device plays the role of control.

In this case, *D* = (m ÷ V) (2V_1_) where V and V_1_ are in mL, m is in mg and D is in mg/L.

- Stir the mixture of water to be clarified and the coagulant contained in each beaker of the test jar device for 30 min then leave the mixtures to rest for 1 hour.
- The average doses of coagulant introduced into the 6 beakers of the jar test device are equal to 0 mg/L, 20 mg/L, 30 mg/L, 40 mg/L, 50 mg/L, 60 mg/L and the percentages of reduction of turbidity of clarified water were respectively on average equal to 27.5 %, 87.5 %, 93.75, 95 %, 93.75 %, 90 % (Fig. 1).
- The dose of alum which allowed the greatest reduction in turbidity is on average 40 mg/L.

The results of this research work have shown that the coagulant produced rom the seeds of Moringa oleifera can be used effectively for the treatment of lake water. A dose of 0.7 mg / L of seeds of this coagulant was sufficient to obtain a residual turbidity of 2 NTU, while 40 mg / L of aluminum sulfate is required to obtain the same result.

The residual turbidity obtained after the treatment of lake water with the coagulant produced was in accordance with the drinking water standard according to the World Health Organization (WHO) (less than 5 NTU).

The pH of the treated water is very important because drinking water must have a pH between 6.5 and 8.5 according to the WHO. The pH of the water treated with the coagulant produced is 7.03; while the pH is 5.81 for water treated with aluminum sulfate.

The concentration of total coliforms and intestinal enterococci in the lake water were respectively 500 and 85 per ml of water, before treatment. After treatment with the coagulant produced, using the test jar method, the treated lake water no longer contained total coliforms or intestinal enterococci.

The elimination of microbes in treated water is a function of the initial physicochemical and microbiological characteristics of the raw water and is proportional to the normalization of turbidity [16]. Indeed, the microorganisms being fixed on the particles in suspension, their sedimentation also causes that of the microorganisms. These results are in agreement with those obtained by other authors [17,18].

## Conclusions

This study made it possible to produce a natural coagulant from moringa oleifera seeds by treating them with soxhlet then with sodium chloride solution. The coagulant produced made it possible to clarify lake water with an initial turbidity of 40 NTU. After treatment, water turbidity was reduced by 95 %. The novelty of this method is that the seeds of Moringa oleifera are free of oil and mineral salts making the coagulant obtained more effective for the treatment of low turbidity water. Water treatment with this coagulant is inexpensive and does not generate non-biodegradable residues like chemical processes. This coagulant produced by this new method from moringa oleifera seed is an alternative to aluminum sulfate coagulant for water treatment.

## CRediT authorship contribution statement

**Parfait Sagnon Hounsinou:** Conceptualization, Formal analysis, Methodology, Software, Visualization, Writing – original draft, Writing – review & editing. **Fidèle Mahoudo Assogba:** Methodology, Visualization, Formal analysis, Writing – review & editing. **Miriam Hounsinou:** Conceptualization, Methodology, Software, Writing – original draft. **Julien Adounkpè:** Investigation, Methodology, Validation. **Lyde Sèwèdo Arsène Tomètin:** Methodology, Visualization, Formal analysis. **Achille Comlan Dedjiho:** Methodology, Visualization, Formal analysis. **Waris Kéwouyèmi Chouti:** Investigation, Methodology, Validation. **Daouda Mama:** Supervision, Methodology, Validation. **Joachim Djimon Gbénou:** Supervision, Methodology, Validation. **Eléonore Yayi Ladekan:** Supervision, Methodology, Validation.

## Declaration of Competing Interest

The authors declare that they have no known competing financial interests or personal relationships that could have appeared to influence the work reported in this paper.

## Data Availability

The authors are unable or have chosen not to specify which data has been used.
